# Exploring the Use of Hybrid Closed‐Loop Systems in People With Type 1 Diabetes and Their Partners: A Qualitative Evaluation From the NHS England Pilot

**DOI:** 10.1155/jdr/1997861

**Published:** 2026-02-16

**Authors:** Jennifer Hagan, Tomás P. Griffin, Radhika Chauhan, Pratik Choudhary, Thomas S. J. Crabtree, Dawn Ackroyd, Emma G. Wilmot, Parth Narendran, Zosanglura Bawlchhim, Jackie Elliott, Michelle Hadjiconstantinou

**Affiliations:** ^1^ Diabetes Research Centre, College of Health Sciences, University of Leicester, Leicester, UK, le.ac.uk; ^2^ Centre for Diabetes, Endocrinology and Metabolism, Galway University Hospitals, Galway, Ireland, hse.ie; ^3^ School of Medicine, University of Galway, Galway, Ireland, universityofgalway.ie; ^4^ Division of Psychology, School of Applied Social Sciences, Faculty of Health and Life Sciences, De Montfort University, Leicester, UK, dmu.ac.uk; ^5^ Department of Diabetes & Endocrinology, Royal Derby Hospital, University Hospitals of Derby and Burton NHS Trust, Derby, UK, nhs.uk; ^6^ School of Medicine, Faculty of Medicine and Health Sciences, University of Nottingham, Nottingham, UK, nottingham.ac.uk; ^7^ Leicester Diabetes Centre, University Hospitals of Leicester, Leicester, UK, nhs.uk; ^8^ Department of Immunology and Immunotherapy, University of Birmingham, Birmingham, UK, birmingham.ac.uk; ^9^ CEDAR Centre, Department of Diabetes, Royal Surrey NHS Foundation Trust, Guildford, UK; ^10^ Diabetes and Endocrine Centre, Sheffield Teaching Hospitals, Department of Oncology and Metabolism, The University of Sheffield, Sheffield, UK, sheffield.ac.uk

**Keywords:** experiences, hybrid closed-loop, qualitative, Type 1 diabetes

## Abstract

**Aims:**

The NHS England hybrid closed‐loop (HCL) insulin pump pilot offered people living with Type 1 diabetes (PWT1Ds) access to HCL therapy. Outcomes demonstrated the glycaemic benefits of HCL. Our study explored the views, experiences and impact of HCL on users and their partners′ daily life.

**Methods:**

A total of 14 PWT1Ds and 12 partners of PWT1Ds who participated in the NHS HCL pilot took part in semistructured interviews via telephone/video call. Topics explored included the effect of the HCL system on glucose levels, time spent managing diabetes, daily life and challenges with the systems. Interviews were audio‐recorded and transcribed. Data was analysed using inductive thematic analysis and then mapped onto an adapted Optimal Health Wheel (OHW) framework encompassing four relevant domains: (i) emotional, (ii) intellectual, (iii) social and (iv) physical.

**Results:**

Ten subthemes relating to the impact or experience of using HCL emerged—knowledge and previous experience, time/trial and error, building trust, impact on mental wellbeing, impact on physical health, impact on diabetes management, impact on lifestyle, impact on work, impact on relationships and need for support. PWT1Ds and partners reported multifaceted physiological and psychosocial benefits of using HCL systems. While technical difficulties and initial learning hurdles were acknowledged as barriers to HCL use, facilitators such as previous experience and trial and error helped overcome these issues.

**Conclusions:**

PWT1Ds and their partners endorsed the use of HCL systems, despite challenges, due to the impactful benefits to their lives. To ensure future successful implementation of HCL, users should be offered appropriate training and access to support to help build trust. These findings underscore the potential of HCL systems in T1D treatment.

## 1. Introduction

The management of Type 1 diabetes (T1D) presents an ongoing burden, requiring people living with Type 1 diabetes (PWT1Ds) to measure their glucose and inject insulin multiple times a day to respond to a number of predictable and unpredictable factors [1, 2]. This unrelenting burden of self‐care contributes to the high rates of depression and distress seen in T1D [3, 4]. Diabetes‐specific distress, which refers to the emotional burdens and worries from managing diabetes [5], is particularly problematic, especially in those with higher glucose levels. This extends to partners and family members of PWT1Ds who often also report increased anxiety and reduced quality of life [6].

Diabetes technology can reduce the burden of living with diabetes and improve quality of life [7, 8]. Hybrid closed‐loop (HCL) systems consisting of an insulin pump that can adjust insulin delivery every few minutes based on data from a continuous glucose sensor (CGM) have shown improved glycaemic control, high treatment satisfaction and reduced diabetes distress in randomised studies [9]. In 2021, National Health Service (NHS) England funded a real‐world pilot roll‐out of commercially available HCL systems [10].

Results from the pilot showed a large improvement in glucose control, with associated reductions in diabetes distress [11], but further research into the impact of HCL on daily life and experiences of HCL use in the NHS real‐world setting is lacking—specifically qualitative research. Existing qualitative studies have explored HCL use in adults with T1D, including expectations, benefits, problems, experiences and impact on daily life [12–15]. However, in some clinical trials, study conditions are not representative of real‐world HCL use (such as participants staying in hotels) [12], some only explore short‐term use of HCL [13, 15], and others only investigate one HCL system [14]. Additionally, many existing qualitative studies explore precommercial, prototype products, meaning there may be differences in systems and subsequent participant experiences compared with current commercial systems.

Furthermore, few studies interview partners of HCL users. In the only qualitative study to date exploring partner experiences of HCL use in women living with T1D, Quintanilha et al. examined postpartum use of Medtronic systems (MiniMed 670G/770G) [16]. They found that both partners and PWT1Ds shared frustrations about the algorithm maintaining glucose levels higher than desired, perceiving the device as overly conservative. Partners nevertheless valued the marked reduction in hypoglycaemia, particularly overnight, which eased the burden of newborn care. Some partners also reflected on the transition to auto mode with interest, noting that it would be ‘interesting to see’ how the system assumed a degree of control previously exercised by the woman herself, highlighting the interpersonal adjustments that accompany technology adoption [16].

Living with T1D places strain on intimate relationships, largely due to the constant threat of severe hypoglycaemia, which partners find distressing to witness and manage [6]. Partners frequently report anxiety, disrupted sleep and feeling underprepared to support the person with diabetes, contributing to role strain and psychosocial burden, including diabetes distress [6]. At the same time, positive partner involvement consistently predicts better outcomes for PWT1Ds, including improved glycaemic control, lower diabetes distress and enhanced quality of life [17, 18]. Given that partners of PWT1Ds are often significantly involved in day‐to‐day diabetes care, HCL systems are likely to affect their lives as well as those of users. Their perspectives are therefore essential for understanding how HCL shapes everyday life, shared responsibilities and relationship dynamics. Gaining partners′ views offers important insights into both the benefits and challenges of HCL for users and their partners.

Considering this gap in the literature and current plans for HCL implementation, the present study was aimed at the following:

1. Exploring the views and experiences of PWT1Ds using a HCL system and their partners.
2. Exploring the impact of HCL on daily life and functioning amongst PWT1Ds and partners.

Specifically, we interviewed individuals who used a commercial HCL system long‐term (over 6 months), in real‐world conditions (the NHS HCL pilot), and their partners. Although it was not possible to compare usability across devices, the invitation to the interviews was open to individuals using any of the systems available in the NHS England HCL pilot.

## 2. Methods

### 2.1. Study Design and Setting

This qualitative study was part of a mixed‐methods evaluation study, exploring the impact of HCL systems on patient‐reported outcomes in PWT1Ds and their partners [19].

The study received Research Ethics Committee (REC) favourable opinion and HRA approval from the West Midlands–Black Country REC (21/WM/0245).

The Reflexive Thematic Analysis Reporting Guidelines [20] were considered in reporting this study to facilitate methodological coherence.

### 2.2. Participants

#### 2.2.1. Recruitment of PWT1Ds

PWT1Ds participating in the NHS HCL pilot at 31 adult sites in England [10] were invited to take part in the qualitative study. To be eligible for the pilot, the PWT1Ds had to (i) be using an insulin pump and intermittently scanned continuous glucose monitoring for more than 3 months and (ii) have a recent (within 3 months) HbA1c ≥ 69 mmol/mol. Those who expressed interest gave electronic informed consent built into www.onlinesurveys.ac.uk and indicated whether they would participate in a one‐off semistructured interview.

Purposive sampling was used to obtain a sample representative of the wider NHS England HCL pilot in terms of age, gender, ethnicity and HCL system. PWT1Ds were invited to participate, and interviews were arranged. Interviews took place between June 2022 and July 2023.

#### 2.2.2. Recruitment of Partners

PWT1Ds were also asked whether their partners could be contacted about the study. Partners who expressed interest gave electronic informed consent and indicated whether they would participate in the one‐off interview. The qualitative researcher contacted the partners to arrange interviews. These took place between June 2022 and March 2023.

For both PWT1Ds and partners, recruitment continued until the sample held sufficient ‘information power’ [21] to generate new insights around the impact and experiences of HCL. The study aims, sample specificity and the quality of interviews were considered when determining sufficient information power.

### 2.3. Data Collection

Semistructured interviews were conducted with PWT1Ds between June 2022 and July 2023 and with partners between June 2022 and March 2023. Topic guides with open‐ended questions steered discussions and follow‐up questions explored emerging topics. This flexibility facilitates participant‐led discussions, enabling inclusion of topics participants deem important, which may have been overlooked in the topic guide [22].

Topic guides were developed using the literature and expertise of the team (a qualitative researcher and consultants with expertise in T1D and diabetes technology). Three people with lived experience of T1D shared their thoughts on the questions. Two topic guides were developed: one for PWT1Ds and one for partners. Questions covered were as follows: effect of HCL on glucose control, managing diabetes, daily life (wellbeing, sleep, relationships, work and leisure) and challenges/issues with HCL (see Appendices 1 and 2). Questions within the partner topic guide are aimed at exploring the impact on the partner′s lives in addition to the PWT1Ds′, although it was noted that many partners predominantly spoke of the PWT1Ds′ experiences.

Interviews were facilitated by qualitative research assistants—the majority by R.C., and two by M.C. The interviewers had no prior relationship with participants and wrote field notes and personal reflections following interviews. Both interviewers had experience conducting semistructured interviews and knowledge of T1D. Interviews with PWT1Ds ranged between 24 and 82 min, whilst interviews with partners ranged between 24 and 63 min. Interviews were conducted via telephone or video call (Microsoft Teams). Calls were audio‐recorded and transcribed verbatim by an independent transcriber.

### 2.4. Data Analysis

Following completion of data collection, data analysis commenced. NVivo 12 (qualitative indexing software) was used to facilitate data management and analysis. Reflexive thematic analysis was used to analyse the data [23]. Data were coded by J.H. (a qualitative research assistant with a background in health psychology and experience conducting thematic analysis) and critically discussed with a senior qualitative researcher (with significant experience and knowledge of thematic analysis and diabetes research) (M.H.) to facilitate reflexivity. During these discussions, codes were reviewed and the researchers reflected on how the data were being interpreted. Relevant, meaningful codes relating to the views and experiences of PWT1Ds using a HCL system and the impact of a HCL system on daily life and functioning were organised into themes/subthemes by J.H. and then iteratively refined. Semantic themes were generated, capturing explicit meanings of the data. The researchers who conducted the analysis did not conduct the interviews due to changes in staff. The research assistant who conducted the analysis (J.H.) undertook significant familiarisation with the data, read the interviewers′ field notes and attended training to develop their knowledge and understanding of diabetes technology and T1D.

The constructionist paradigm underpinned the analysis, focusing on understanding how social context might impact an individual′s account of experiences and meanings [23]. The initial analysis was inductive; themes were generated exclusively from the data, not based on researcher preconceptions [23]. Data was subsequently deductively mapped onto an adapted Optimal Health Wheel (OHW) [24], a holistic model of health, encompassing six domains contributing to achieving optimal satisfaction with health—emotional, physical, intellectual, social, occupational and spiritual [25]. The OHW has been successfully used as a data analysis framework in previous qualitative diabetes research [26] and was used in the current study due to its relevance to optimal health within diabetes. In this analysis, four out of six domains were relevant. The occupational and spiritual domains were not deemed relevant due to a lack of codes relating to these domains.

## 3. Results

In total, 177 PWT1Ds were invited to participate in the mixed‐methods study. Of these, 125 completed the quantitative component and 102 consented to be contacted for the qualitative interviews. Additionally, 57 partners of PWT1Ds were invited; 33 completed the quantitative component and 20 consented to be contacted for the qualitative interviews. A total of 26 participants (14 PWT1Ds and 12 partners of PWT1Ds) took part in the interviews. Five out of 12 partners were partners of the interviewed PWT1Ds.

Of PWT1Ds, 57.1% were male and 78.6% White British. The mean age of participants was 47.3 (12.9) years. Duration of HCL use was between 6 and 9.5 months at interview. The majority of participants (64%) were using the Medtronic 780G HCL system. This reflects the demographics of the overall NHS England pilot sample [11]. Of partners, 66.7% were male and 91.7% White British; their partners (with T1D) had a duration of HCL use between 7 and 15.5 months at the time of the interview.

Participant demographic characteristics are presented in Tables 1 and 2.

**Table 1:** Characteristics of people with T1D.

Characteristic	Category	Total (*N* = 14)
		*n* (%)
Age	20–30 years	2 (14.3)
	31–40 years	3 (21.4)
	41–50 years	2 (14.3)
	51–60 years	5 (35.7)
	60+ years	2 (14.3)

Gender	Male	8 (57.1)
	Female	6 (42.9)

Ethnicity	White—British	11 (78.6)
	White—Other	1 (7.1)
	Asian	0 (0)
	Mixed	2 (14.3)

Marital status	Married/civil partner	11 (78.6)
	Single	2 (14.3)
	Divorced	0
	Other	1 (7.1)

Employment status	Full‐time	8 (57.1)
	Part‐time	1 (7.1)
	Student	1 (7.1)
	Retired	1 (7.1)
	Other	3 (21.4)

Insulin pump prior to hybrid closed‐loop	Roche Accu‐Chek Insight	3 (21.4)
	Medtronic 640G	9 (64.3)
	Omnipod	1 (7.1)
	DANA RS	1 (7.1)

Hybrid closed‐loop system	Tandem Control IQ	2 (14.3)
	Medtronic 780G	9 (64.3)
	CamAPS FX	3 (21.4)

Duration of HCL use (months)^a^		7 (6–9.5)

^a^Median (minimum–maximum).

**Table 2:** Characteristics of partners of people with T1D.

Characteristic	Category	Total (*N* = 12)
Age	20–30 years	0 (0)
	31–40 years	2 (16.7)
	41–50 years	3 (25)
	51–60 years	5 (41.7)
	60+ years	2 (16.7)

Gender	Male	8 (66.7)
	Female	4 (33.3)

Ethnicity	White—British	11 (91.7)
	White—other	0 (0)
	Asian	0 (0)
	Mixed	1 (8.3)

Marital status	Married/civil partner	10 (83.3)
	Single	0 (0)
	Divorced	1 (8.3)
	Other	1 (8.3)

Employment status	Full‐time	9 (75)
	Part‐time	1 (8.3)
	Student	0 (0)
	Retired	0 (0)
	Other	2 (16.7)

Duration of partners′ HCL use (months)^a^		14 (7–15.5)

^a^Median (minimum–maximum).

**Figure 1:**
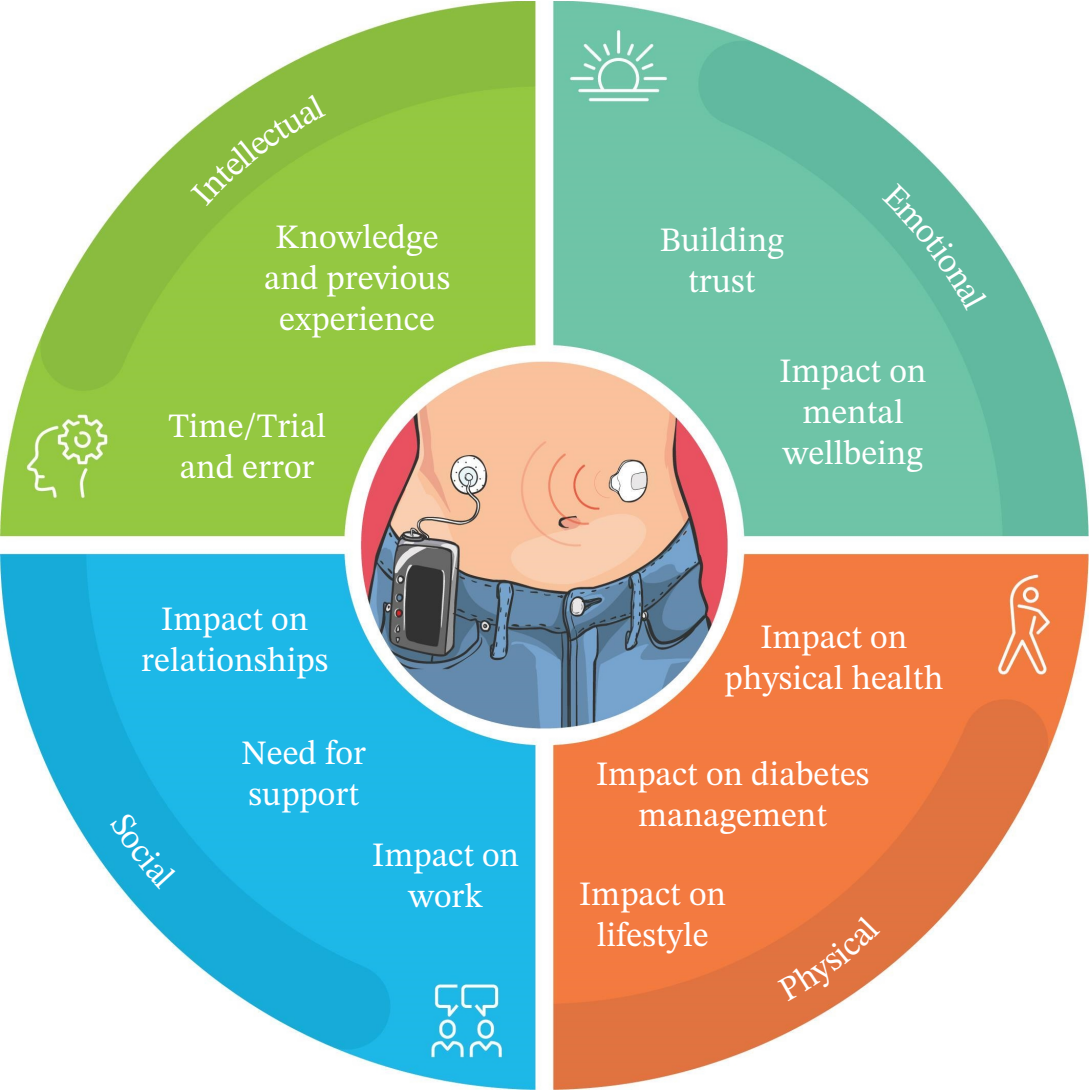
The four relevant dimensions of the OHW, with the aligned subthemes relating to experiences and impact of HCL.

The analysis produced 10 subthemes relating to the impact or experience of using HCL, which align with 4 dimensions of the OHW. Figure 1 depicts the relevant dimensions and corresponding subthemes in the adapted OHW framework: (i) emotional, (ii) intellectual, (iii) social and (iv) physical.

### 3.1. Emotional

#### 3.1.1. Building Trust

In early stages of HCL use, PWT1Ds reported a lack of trust in HCL, causing concerns and reluctance to use the system. Some doubted the accuracy and reliability of HCL and feared technology failures.

I just didn′t actually fancy leaving it to a pump to actually keep everything under control sort of thing. I didn′t trust the technology if I′m honest. (PWT1D, male, 59, Medtronic 780G)

Some concerns were reinforced for those who faced technical challenges, for example, cannula and sensor failures, connectivity and calibration problems and service outages.

Sometimes you have some crazy numbers because the needle, there′s something wrong with the needle or the canula or something. (PWT1D, male, 47, CamAPS FX)

Despite technology challenges, participants reported that concerns were often overcome following continued HCL use. Seeing visual results, such as patterns of glucose and graphs, was beneficial for many. This built trust as they could see the system was working.

You can see the HbA1Cs coming down… that has given me the confidence to properly trust everything because you′ve seen improvements. (PWT1D, female, 31, Medtronic 780G)

These visual results also acted as positive reinforcement, helping participants feel more confident in managing their diabetes using the system and pleased with their progress.

It tells you when you′re doing well and the higher the number the better of course. So there is a definite feeling of achievement on reaching 100 which is a real positive thing because it then makes you feel much more positive about diabetes as a whole and the system and so on. (PWT1D, male, 53, Medtronic 780G)

Partners also expressed similar worries during the initial adjustment period and had concerns about the reduced level of input required from both their partner and themselves. However, in line with PWT1Ds, partners also gained trust after seeing the positive impact of the device on their partners′ diabetes management.

Just trusting the device, that was quite difficult to do at first because I think before you had to be doing everything but I think that′s it, just stepping back and letting it do its thing. Because we kept thinking that we needed to intervene but again, we left it and then we were just amazed at how quickly it turned it around and was able to get control of her blood sugar. (Partner, female, 35, Tandem Control IQ)

#### 3.1.2. Impact on Mental Wellbeing

PWT1Ds highlighted the impact of HCL on their mental health. They described having fewer thoughts about diabetes, feeling more hopeful and positive and experiencing reduced low mood, frustration and self‐consciousness.

I feel more energised, more engaged, I feel happy like properly for the first time in a long like you know, there′s not that dark burden in the back of my mind. (PWT1D, female, 37, Tandem Control IQ)

Peace of mind and feeling ‘normal’ was commonly reported. Participants noted various factors contributing to these improvements in mental wellbeing, such as stabilised glucose leading to more balanced moods.

I am so much more chilled out now… whereas before – and maybe that was blood sugar related, I don′t know, but I felt snappy all the time, like a bit aggressive and a bit angry I guess… I don′t any more. (PWT1D, female, 37, Tandem Control IQ)

Furthermore, some felt that an increased sense of normality due to newfound flexibility and independence in their day‐to‐day lives resulted in improved quality of life and, thus, mental wellbeing.

This just allows you to be able to live and enjoy life without having to be dictated to by the diabetes. (PWT1D, female, 31, Medtronic 780G)

Many of these benefits were also reported by partners.

…because she′s more positive about it I am as well. I′m not so kind of quietly worried about it. (Partner, female, 35, Tandem Control IQ)

However, certain anxieties relating to managing the system (including fear of technology failure and supply issues) emerged in some cases.

The peripherals management does concern me, in terms of making sure that we′ve got enough stuff at all times. (Partner, male, 48, CamAPS FX)

### 3.2. Intellectual

#### 3.2.1. Knowledge and Previous Experience

Some participants found the learning curve of HCL challenging, reporting information overload, self‐learning and difficulty understanding the system. Device failures were described as particularly difficult to cope with, as individuals lacked understanding of the issue and how to fix it.

It was tough at the start. It was completely different. It was a big learning curve and I noticed when I went to like the training session for it, there were a few people that were struggling to grasp it. (PWT1D, male, 26, Medtronic 780G)

In contrast, knowledge and previous experience of diabetes technology facilitated HCL use. Individuals described transferring their knowledge from previous pumps and applying their learnings to HCL. They felt this made adjusting to HCL easier and less daunting and improved their confidence.

I had already been using a pump so this is basically the same as before with some extra functions. So the basics of using a pump weren′t an issue for me. I was familiar with that. (PWT1D, male, 53, Medtronic 780G)

#### 3.2.2. Time/Trial and Error

Spending time getting used to HCL and using trial and error was key in improving experiences. Over time, confidence grew, and PWT1Ds tweaked settings (with the support of healthcare professionals) to meet their individual needs, for example, creating different profiles for different levels of activity and altering carb ratios. This enabled individuals to overcome knowledge and understanding barriers.

You′ve got to be open, willing, put the work in at the beginning – you′re talking like a month, two months of spending the time to learn it, understand it, playing with it, trial and error and then suddenly it just all falls into place and everything just works. (PWT1D, female, 37, Tandem Control IQ)

### 3.3. Social

#### 3.3.1. Impact on Relationships

Various positive impacts on relationships were reported. Partners felt more reassured, were less involved in diabetes management and experienced reduced frustration, fewer arguments and more pleasant interactions:

[partner feels] Less like a parent, more like a wife so that′s a positive thing. (Partner, female, 36, Medtronic 780G)

Despite this, some drawbacks were reported such as alarms bothering partners and interrupting intimacy.

As I say, the only thing that I noticed was…um…kind of intimate activity…so my husband′s found it a bit annoying at times, it bleeps, because it wants blood sugar in the middle of whatever! (PWT1D, female, 31, Medtronic 780G)

HCL use also impacted PWT1Ds′ wider friends and family. Participants felt like less of a burden on family members due to increased independence and reduced reliance on them. Family members were also less involved in their diabetes management and worried less, as they were more confident that the PWT1Ds had stable glucose levels due to the HCL.

It′s turned my family′s life round. It′s given them peace of mind to enable them to go and do what they need to do rather than keep going ‘Is she all right? What is she doing? What is that?’ (PWT1D, female, 58, Medtronic 780G)

For some PWT1Ds, using a HCL system contributed to increased diabetes awareness amongst others around them. Participants described the system sparking conversations about diabetes, others asking more questions, and feeling more open discussing their diabetes.

It [HCL] is making type 1 diabetes more visual and more out there and people are starting to understand a bit more about it. (Partner, male, 48, CamAPS FX)

#### 3.3.2. Impact on Work

Participants highlighted numerous improvements to their working lives since beginning HCL. For example, PWT1Ds felt less reliant on colleagues, were more focussed and reliable, had more energy to work and could work to more flexible schedules. Participants reported reasons for these benefits including fewer incidents of hypoglycaemia at work, not having to stick to strict lunch schedules and being off sick less.

…she′s having to rely on people there less and people a bit less kind of feeling like they′re having to cover for her whilst she′s ill. (Partner, male, 46, Tandem Control IQ)

Increased safety was also discussed in relation to driving and lone working, due to a reduced risk of hypoglycaemia.

I′m able to get on with doing my job and, you know, it′s kind of, like, a bit of security almost. (PWT1D, female, 31, Medtronic 780G)

#### 3.3.3. Need for Support

Support from others contributed to PWT1Ds′ positive experiences using HCL. Participants stressed the importance of having sufficient guidance and training and having access to help when experiencing issues.

With the support of – with Dr [Name 2] especially and [Name 4] especially – I feel I′m getting there, I′m more confident, I know what I′m doing. (PWT1D, female, 53, CamAPS FX)

Support from friends/family, such as assistance attaching devices, managing supplies and online support groups, was also valued by participants. This extra support was seen more as an added positive and acted as a backup, rather than absolutely necessary, except in atypical circumstances such as the PWT1Ds and having restricted movement.

I do all the cannulas and things but he (PWT1D′s son) can do as well and has done…that always helps. (Partner, male, 59, Medtronic 780G)

Although most participants received adequate support, others felt they were lacking support, which was challenging. Some felt unable to access support, experienced inadequate communication from healthcare providers and felt that training was insufficient.

My diabetes team at the hospital I think are very stretched with what they′re able to offer me support wise with this. (PWT1D, female, 47, Medtronic 780G)

### 3.4. Physical

#### 3.4.1. Impact on Physical Health

HCL use had an impact on the general health of PWT1Ds; participants reported better resistance to illness and fewer diabetes‐related symptoms (i.e., problems with eyes and feet). Many participants also reported weight loss, which they previously struggled to achieve, and highlighted the possible minimisation of long‐term health impacts.

You don′t have to be having your eyesight going all blurry and fizzy. Your finger ends aren′t all like pins and needles. You′ve not got that joint pain. You′ve not got the tiredness, fatigue. (PWT1D, female, 58, Medtronic 780G)

In particular, the positive impact on glucose was highlighted by all participants; after commencing HCL use, PWT1Ds experienced reductions in glucose, greater time in range and fewer extreme highs/lows. Many participants also reported reduced hypoglycaemia, which became easier to manage.

I just had an HbA1c done a couple of weeks ago, and it is about the lowest, the best HbA1c I′ve ever had in my life. (PWT1D, male, 53, Medtronic 780G)

For some, hypoglycaemia became more frequent; however, they perceived this as generally milder and due to the adjustment to overall lower glucose, having previously had a much higher blood glucose. Hence, this was not viewed as a particularly negative impact but something to adapt to.

I am still having more lows than I did before but I was barely having any lows before I started [HCL] because I was running high so often… I′m fine with it…I can tell when they′re coming, I can manage them. I never suffer for ages. (PWT1D, male, 26, Medtronic 780G)

#### 3.4.2. Impact on Diabetes Management

Participants outlined positive impacts of HCL use on diabetes management. Overall, PWT1Ds experienced reduced burden due to the ease and convenience of HCL—management became easier, required less attention and reduced involvement from partners.

…this [HCL] makes it [diabetes], I was going to say manageable. It′s not even manageable because you don′t really have to manage it. Yes, I can′t help but praise this. (PWT1D, male, 47, CamAPS FX)

Furthermore, PWT1Ds and their partners felt more confident and knowledgeable in diabetes management following HCL use.

However, some practical and technical challenges impacted participants′ diabetes management. Some found alarms, insulin refills and calibrations occurred at inconvenient times, experienced inconsistency in obtaining supplies and felt the device would easily get caught on things.

The other day I caught it on the door, it ripped it completely out of my skin, I mean, it kills, you know what I mean, it hurts for like 30 seconds, and you′re like arrgghhh why have I got this stupid pump! (PWT1D, male, 36, Tandem Control IQ)

#### 3.4.3. Impact on Lifestyle

Participants discussed the impact of HCL use on various components of their lifestyle, including diet, exercise and sleep.

PWT1Ds felt HCL facilitated dietary freedom, enabling flexibility in the type and quantity of food they eat, and the rigidity of their mealtimes. Participants reported greater spontaneity when eating and experienced fewer food‐related consequences such as spikes in blood sugar.

I′m probably less rigid about eating than I was… now if I′m not hungry I′m more inclined not to eat, if that makes sense which, as I′m always trying to lose weight not such a bad thing. (PWT1D, female, 57, Medtronic 780G)

In relation to sleep, PWT1Ds reported reduced hypoglycaemia at night due to HCL, resulting in being woken up less. Hypoglycaemia at night also became quicker and easier to manage, meaning participants spent less time awake. Partners also experienced these benefits, as they were woken up less by the PWT1Ds.

…he′s having less hypos so I′m not woken up as much. (Partner, female, 36, Medtronic 780G)

Conversely, some participants felt HCL had no impact on their sleep or experienced more disrupted sleep due to alarms waking them and/or their partners up throughout the night.

Oh that′s the prime time, yeah definitely beeping at three or four in the morning, it wakes everybody up in the house. (PWT1D, male, 36, Tandem Control IQ)

Finally, as described by participants, HCL systems allowed PWT1Ds to exercise safely due to a reduced likelihood of hypoglycaemia during exercise. Many felt they were unable to exercise prior to HCL due to fear of hypoglycaemia. Individuals also felt their physical capability had improved since HCL onset, as they could exercise for longer without having to stop due to low glucose levels.

I started going to the gym for the first time…because I knew I had the system and it would stop giving me insulin if my blood sugar started to drop quickly…it′s improved my confidence at trying new things. (PWT1D, female, 57, Medtronic 780G)

Although some highlighted difficulties in contact sports (i.e., rugby), where the system can be accidentally pulled out.

## 4. Discussion

The current study captured the impact of HCL use on people with T1D and their partners within the NHS England Pilot programme, providing a comprehensive overview of their thoughts and experiences whilst exploring the impact of HCL across various domains. Despite challenges, participants endorsed HCL due to the multifaceted benefits to their lives, contributing to overall positive experiences, including improved mental wellbeing, lifestyle benefits and improved diabetes management. These insights add to existing literature by improving understanding of the psychosocial impact of HCL and its effects on daily activities and relationships.

This study is important as it captures the impact of HCL in the real world amongst those who have struggled most with managing their diabetes. Most previous data [13, 15] are from randomised controlled trials that have been shorter term (usually 3–6 months) and with much lower baseline HbA1c compared with that of the participants in the NHS England–funded HCL pilot.

The clinical benefits of HCL established in the current study provided further support of improvements in glycaemic control [11], reported by most participants. Our study also revealed the self‐reported impact of HCL on PWT1Ds′ physical health, including improved resistance to illness, weight loss and fewer diabetes‐related symptoms (i.e., problems with eyes). Participants emphasised perceived minimisation of long‐term health consequences and subsequent hope for the future, which has been previously noted [12]. Given the well‐established relationship between optimism and positive physical health outcomes [27], this is promising.

In addition, impacts on lifestyle were praised; similar to data from randomised controlled trials, reporting less rigid mealtimes, improved sleep quality and reduced hypoglycaemia during exercise [12, 14, 28]. The current study illustrates longer‐term consistency of these effects in the real‐world. In line with existing findings suggesting HCL reduces the burden of diabetes [14, 28], participants also described positive impacts on diabetes management. PWT1Ds and partners felt management became easier and required less attention. These findings are encouraging, considering concerns of technology fatigue in longer duration HCL use [14], although some commonly reported [29] practical and technical challenges were noted, such as inconvenient alarms and calibrations, supply issues and device failures.

Participants also discussed the psychosocial and day‐to‐day impact of HCL. The impact on mental wellbeing was highly valued—participants described more positive moods, fewer thoughts about diabetes and feeling ‘normal’. Similar findings were reported in qualitative studies of shorter duration HCL use [12, 14]; the current study demonstrates longer‐term evidence of these effects. Sources of diabetes distress in PWT1Ds include management, family/friends, fear of hypos and eating [30]. Many participants experienced positive changes in these areas, providing a potential explanation for their improved mental wellbeing.

The current study provides further support for impacts of HCL that have been reported in other populations, such as impact on participants′ working life. PWT1Ds felt less reliant on colleagues, were more focused, energised and flexible at work and felt safer lone working. Positive impacts on work have been previously reported in pregnant women with T1D, who felt HCL enabled them to work for longer [31]. Given the difficulties PWT1Ds face at work [32], these insights are extremely valuable and provide support for a potential treatment for working PWT1Ds.

The impact on relationships (including perspectives of partners of HCL users) was also discussed, which has not previously been addressed. Overall, partners had more pleasant interactions, worried less and were less involved in the PWT1Ds′ diabetes management. These findings demonstrate the widespread impact of HCL, positively affecting those around PWT1Ds, which is important considering the significant distress and burden partners of PWT1Ds can experience [6]. Similar findings have been reported in other family members, such as parents of young children with T1D, who worried less about their child and spent less time delivering diabetes care [33].

### 4.1. Practical Recommendations for HCL Implementation

National Institute of Health and Care Excellence (NICE) recommended HCL systems are offered through the NHS for adults with T1DM who have an HbA1c of 58 mmol/mol (7.5%) or more or have disabling hypoglycaemia, those pregnant or planning pregnancy, and all children and young people [34]. NHS England have now published a 5‐year strategy for HCL implementation across England, based on these recommendations [35], and tens of thousands of PWT1Ds across England are expected to receive a HCL system over the next 5 years [36].

The barriers and facilitators identified have implications for real‐world implementation and continuing development of HCL technology. Firstly, participants highlighted a need for accessible, appropriate support, which has been previously acknowledged [37]. PWT1Ds require guidance and training before beginning HCL to ensure adequate preparation for the potential learning curve and adjustment/trust–building period. Support should be ongoing and available for everyone using HCL for as long as necessary to help navigate issues. A 24‐h helpline could be particularly effective, especially for resolving technical failures. Consistent support would help build trust, mitigate challenges and contribute to a smoother roll‐out with more positive experiences. The data highlighted the personal nature of the benefits that participants and their partners associated with HCL use and how variable these can be.

There are implications for device manufacturers. Various reoccurring technical failures were identified; further refinement of devices would minimise the likelihood of these issues. Practical concerns surrounding the appearance and functionality of devices were raised. As technology develops, the size and durability of devices should be considered (e.g., making them smaller/easier to conceal) to improve acceptability and reduce negative impacts. Although the Medtronic system Guardian G3 sensors required calibration, these have now been replaced by newer, factory calibrated sensors that may result in better user experience.

### 4.2. Strengths and Limitations

This study has numerous strengths. Firstly, participants used HCL in real‐world conditions, meaning their experiences likely represent typical, nontrial HCL use, increasing the transferability of findings. Similarly, participants all used commercially available HCL systems, meaning their experiences can be applied to those receiving HCL systems in practice. Additionally, our sample had used HCL long‐term, 7 months on average for PWT1Ds and 14 months on average for partners. This allowed participants to fully experience HCL, which is key considering the emphasis participants placed on adjustment/learning periods. Finally, by interviewing partners, we gained useful perspectives on the impact and experience of HCL in people around the HCL user.

However, there are limitations to this study. The sample consists of individuals who continued HCL use throughout the NHS HCL pilot and chose to participate in interviews. These individuals likely had more positive experiences and attitudes compared to the overall pilot sample. For example, Crabtree et al. [11] reported 8.8% of pilot participants discontinued HCL; reasons cited include lack of trust/anxiety, problems adjusting to HCL, issues with cannulas/skin site reactions and erratic glucose levels. Hence, future research involving those who discontinue HCL would provide a more accurate representation of HCL barriers and challenges. This could help minimise discontinuation of HCL during the upcoming 5‐year NHS England HCL implementation.

Furthermore, the study sample only included adult HCL users and their partners, despite children also participating in the NHS England pilot. All children and young people will be offered HCL during the NHS England 5‐year implementation plan [35]; therefore, it is important to understand the thoughts and experiences of these individuals, which may differ from adults. Future research exploring this in the NHS England HCL pilot cohort would help highlight potential real‐world benefits or challenges of HCL specific to children and young people.

Finally, due to the exploratory nature of the study, broad interview topics were covered to gain an overall understanding of the experiences and day‐to‐day impact of HCL. Hence, this would benefit from further in‐depth investigation. Future research could focus on specific areas identified by the current study to gain a richer understanding. For example, the impact of HCL on work was highlighted but not thoroughly discussed, and only 57% of participants with T1D were employed full time. As many individuals receiving a HCL system in the NHS roll‐out are likely to be of working age, this is important to examine fully.

## 5. Conclusions

This qualitative study highlights the multifaceted, impactful benefits HCL can have on PWT1Ds and their partners. It highlighted the range of benefits that were very personal and individual and included not only clinical benefits but also benefits to daily life, including lifestyle, emotional and social factors, underscoring the potential value of HCL. Possible challenges and barriers associated with HCL use were also identified, including technical failures, lack of trust and access to appropriate support. The study highlights key factors healthcare providers should consider to mitigate potential challenges in future HCL implementation.

## Funding

This study was funded by JDRF, 10.13039/100022690 (1‐EA‐2021‐0001).

## Conflicts of Interest

P.C. has received personal fees from Sanofi, Lilly, Novo Nordisk, Abbott, Insulet, Dexcom, Medtronic, Vertex, Embecta and Glooko. T.P.G. has received honoraria for speaker fees and/or advisory boards from Novo Nordisk, Dexcom, Abbott Diabetes Care, AbbVie, Bayer Astra Zeneca and Eli Lilly. T.S.J.C. has received personal fees from Abbott Diabetes Care, Dexcom, Insulet, Novo Nordisk, Lilly and Sanofi. E.G.W. has received personal fees from Abbott, AstraZeneca, Dexcom, Eli Lilly, Embecta, Insulet, Medtronic, Novo Nordisk, Roche, Sanofi, Sinocare and Ypsomed. J.E. has received honoraria for speaker fees and/or advisory boards from Abbott, Boehringer, Dexcom, Glooko, Insulet, Lilly, Novo Nordisk, Roche, Sanofi and Ypsomed. J.H., R.C. and M.H. declare no conflicts of interest.

## Data Availability

The data that support the findings of this study are available from the corresponding author upon reasonable request.

## Appendix 1: Interview Topic Guide (PWT1D)

Topic/Prompt Guides for Interviews

Note: The following interview topic guides are flexible tools, with example questions, which are open to revision if new areas of interest arise during the process of data collection. Further, in order to adapt to any logistical factors (e.g., limited time for an interview), it is not essential that each interview should include every line of questioning as detailed below. Depth of exploration of fruitful areas of discussion is more important than complete coverage of all areas in every interview.

In each interview, the interviewer will remind the participant of the project aims and check that they fully understand the participant information sheet and will then take them through the informed consent process. Audio‐recording of the interview will then begin.

Interviews With People With T1D

We are interested in understanding what it is like using a hybrid closed‐loop (HCL) system, and we would like to hear more about your experience.

Introductory questions

1. What has your experience been living with T1D?
2. How would you describe your experience so far managing your diabetes?
3. How much time would you say it takes to manage your diabetes?
4. Have you ever used a hybrid closed‐loop system? If yes, what was it like?
5. What were your expectations when you were asked to use a hybrid closed‐loop system? Prompt: its benefits, daily activities, etc.
6. How reliable did you find the hybrid closed‐loop system?

Prompt:

a. Is reliable/not reliable? Elaborate.

Effects of the HCL on glucose control

1. How did you find using the system? Elaborate.
2. Since using the system, what has your experience been with diabetes management?

Prompts:

a. Has it changed? Elaborate.
b. More/less confident? Elaborate.

3. What has your experience been with incidents of hypos?

Prompts:

a. Experienced more or less
b. Time

4. Do you feel the system has helped you better manage the number of hypos?

Prompts:

a. If yes/no, in what way? Elaborate.

Life outside diabetes (relationships, work, leisure)

1. Since using the system, have you noticed any changes in your mood? If yes, how?
2. Since using the system, have you noticed any changes in your sleep? If yes, how?
3. Have you noticed any changes in your relationship with others? Prompt: How has using the system changed your relationship with others? Specific focus on partner.
4. How would you explain your day‐to‐day life since using the system? Prompt: with work, leisure, etc.
5. Do you think it has changed the way you carry out your daily activities? Elaborate.

Challenges/issues to using the device

1. Would you say there are challenges with the system? Elaborate.

Prompts:

a. Experience with the alarm

2. Have there been any concerns with the hybrid closed‐loop system?

Prompts:

a. Yes/no? Elaborate.

Future of hybrid closed‐loop systems

1. What are your thoughts on closed‐loop systems?
2. If you had the opportunity, would you choose to use it again in the future?
3. Would you recommend it to a friend if it was freely available to them?

Anything not covered?

Is there anything that we have not covered in the interview that you think we should know or think about?

Closing and thanks

Thank them for their time and contribution and check that the participant is still happy for you to use all the information provided and offer the possibility to erase sections of the recording.

## Appendix 2: Interview Topic Guide (Partners)

Topic/Prompt Guides for Interviews

Note: The following interview topic guides are flexible tools, with example questions, which are open to revision if new areas of interest arise during the process of data collection. Further, in order to adapt to any logistical factors (e.g., limited time for an interview), it is not essential that each interview should include every line of questioning as detailed below. Depth of exploration of fruitful areas of discussion is more important than complete coverage of all areas in every interview.

In each interview, the interviewer will remind the participant of the project aims and check that they fully understand the participant information sheet and will then take them through the informed consent process. Audio‐recording of the interview will then begin.

Interviews With Partners

We are interested in understanding what it is like using a hybrid closed‐loop (HCL) system, and we would like to hear more about your experience.

Introductory questions

7. What has your experience been living with someone who has T1D?
8. How would you describe your experience so far with your partner′s diabetes management?
9. What were your expectations when your partner was asked to use a hybrid closed‐loop system? Prompt: its benefits, daily activities, etc.

Effects of the HCL on glucose control

5. How did you find it when your partner first started using the system? Elaborate.
6. Do you feel more/less confident about your partner′s diabetes management since using the system? Elaborate.
7. What has your experience been with your partner′s hypos? Prompts: experienced more or less.
8. Do you feel the system has helped your partner better manage the number of hypos?

Life outside diabetes (relationships, work, leisure)

6. Since using the system, have you noticed any changes in your mood? If yes, how? Prompt: Do you feel less frustrated, worried about your partner′s diabetes management? About your partner′s quality of life?
7. Since using the system, have you noticed any changes in your sleep? In your partner′s sleep? If yes, how?
8. Have you noticed any changes in your partner′s relationship with friends and family?
9. Have you noticed any changes in your relationship with your partner? Prompt: How has using the system changed your relationship with others?
10. How would you explain your partner′s day‐to‐day life since using the system? Prompt: with work, leisure, etc.
11. Do you think it has changed the way your partner carries out your daily activities? Elaborate.

Challenges/issues to using the device

3. What have your concerns been with the HCL system?
4. Have you or your partner experienced any issues with the system? Elaborate.

Future of hybrid closed‐loop systems

4. What are your thoughts of closed‐loop systems?
5. Would you choose for your partner to use it again in the future?
6. Would you recommend it to a friend if it was freely available to them?

Anything not covered?

Is there anything that we have not covered in the interview that you think we should know or think about?

Closing and thanks

Thank them for their time and contribution and check that the participant is still happy for you to use all the information provided and offer the possibility to erase sections of the recording.
